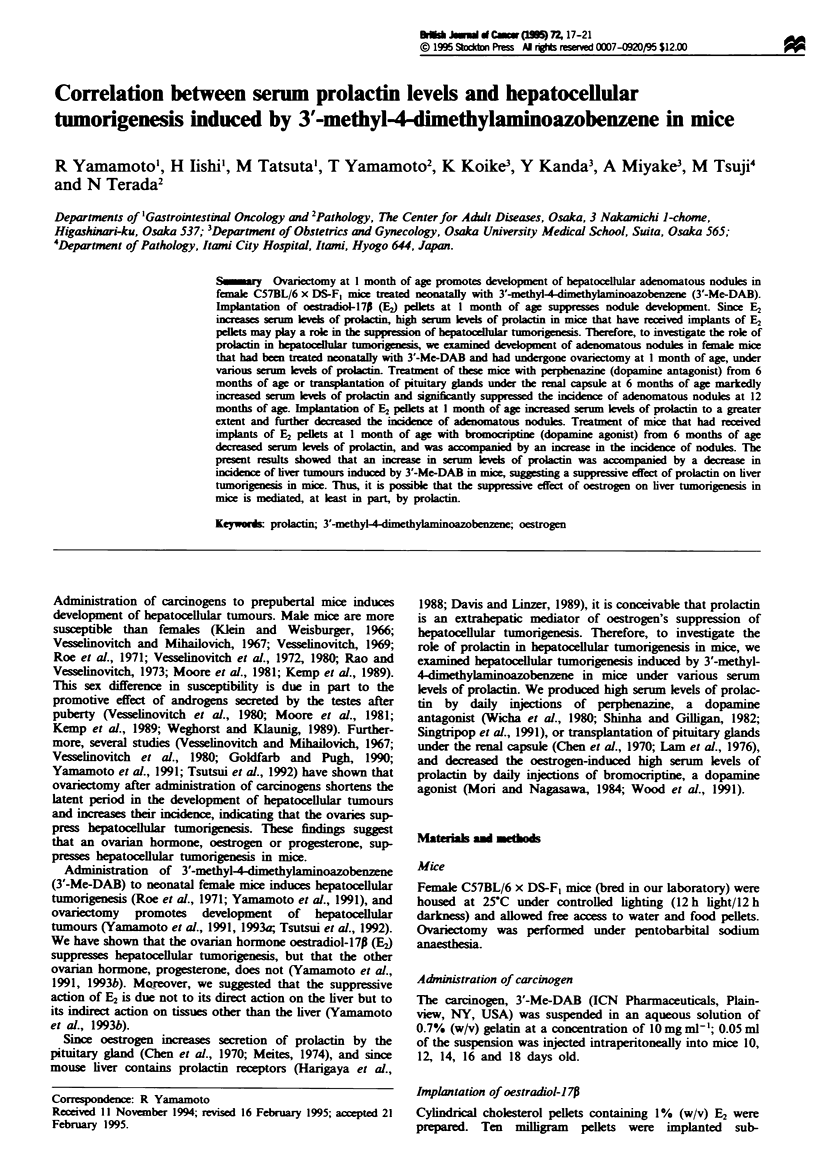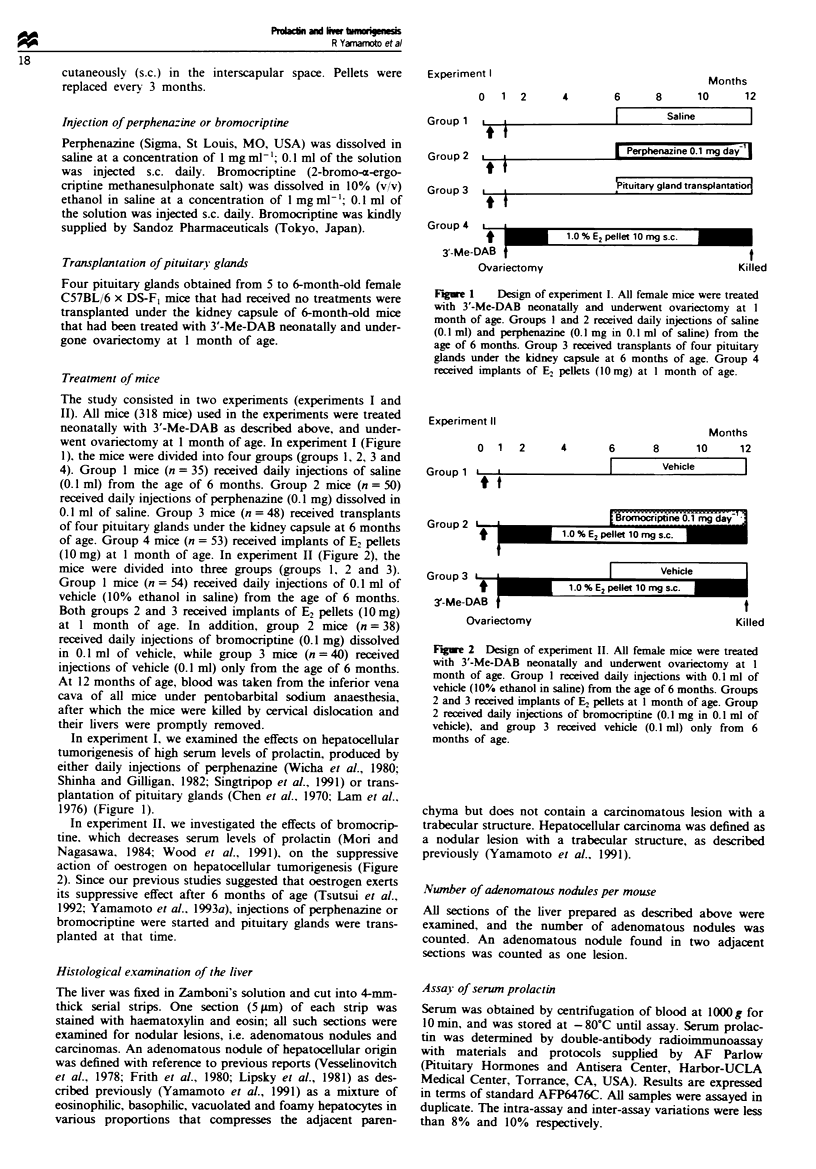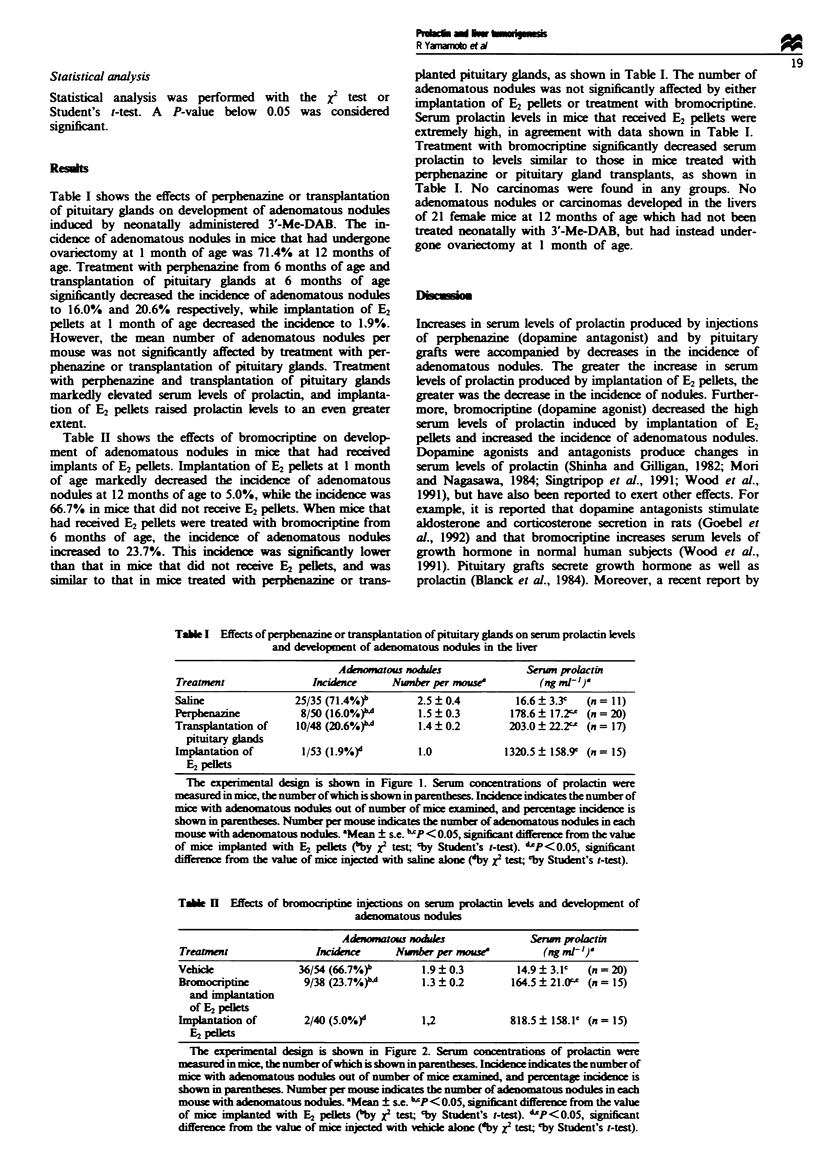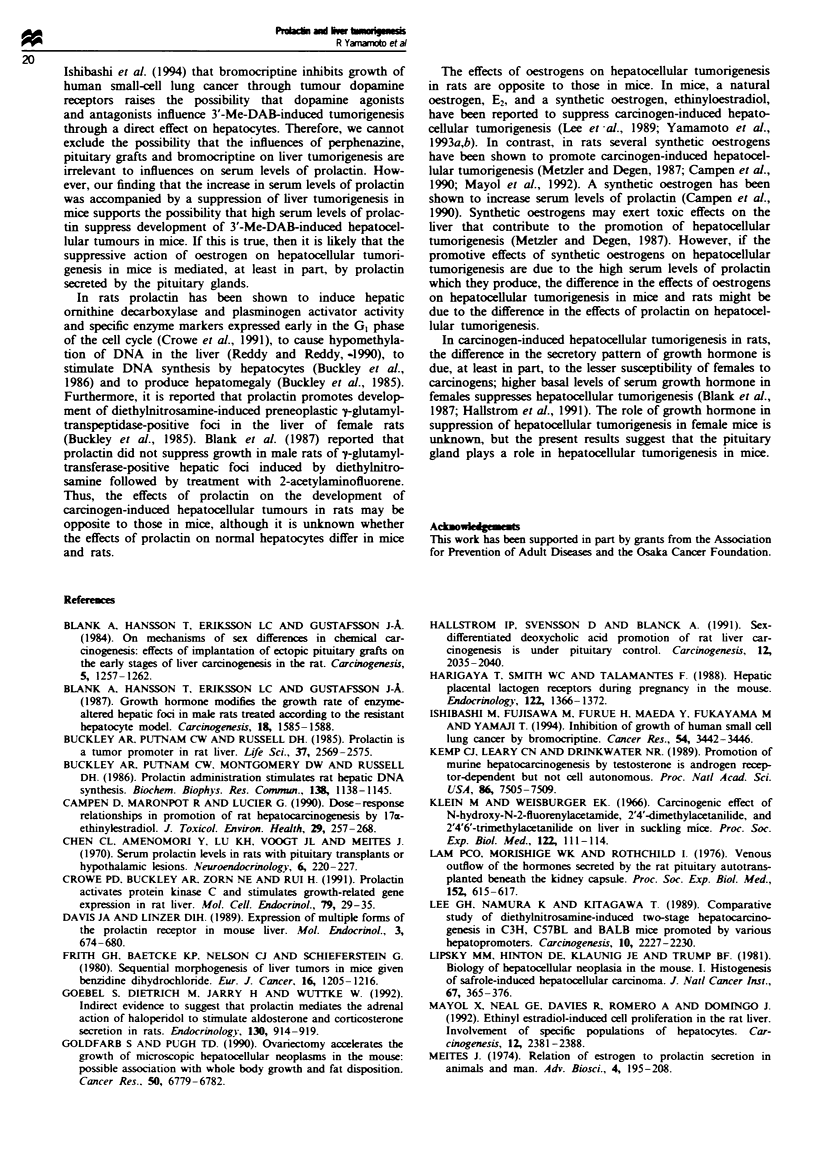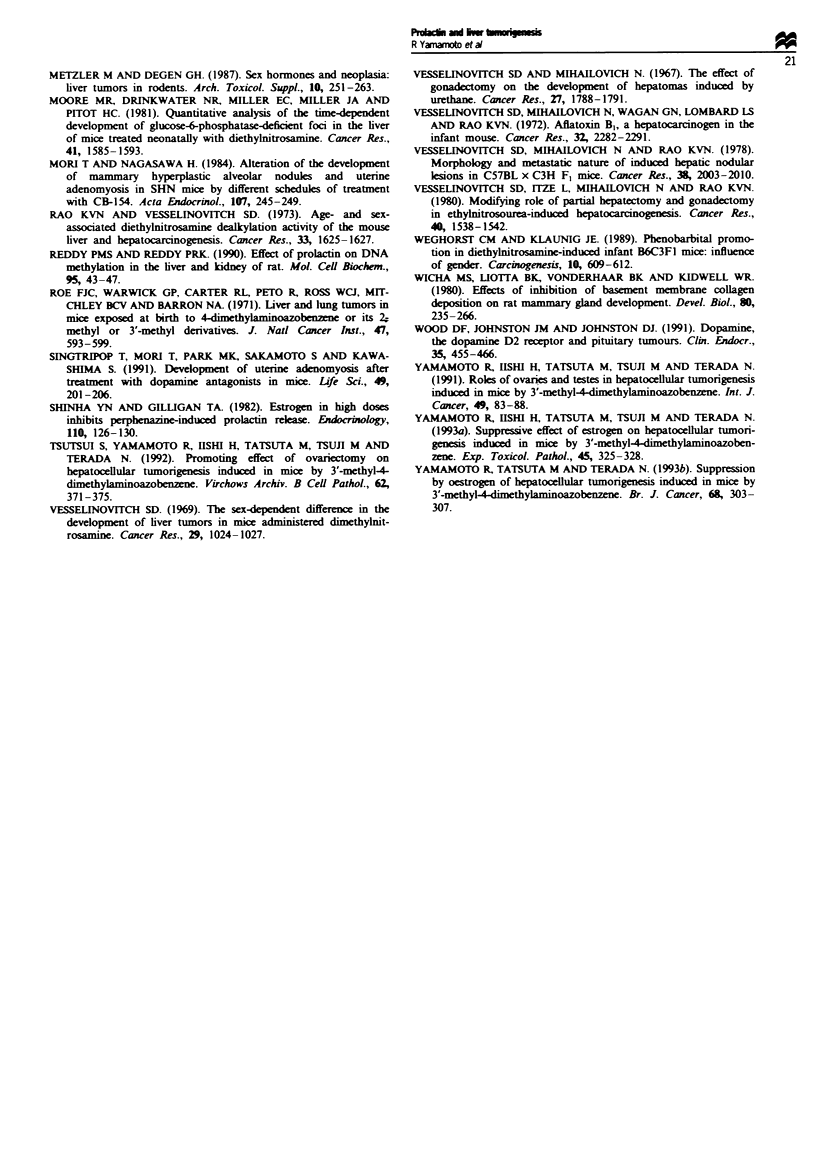# Correlation between serum prolactin levels and hepatocellular tumorigenesis induced by 3'-methyl-4-dimethylaminoazobenzene in mice.

**DOI:** 10.1038/bjc.1995.270

**Published:** 1995-07

**Authors:** R. Yamamoto, H. Iishi, M. Tatsuta, T. Yamamoto, K. Koike, Y. Kanda, A. Miyake, M. Tsuji, N. Terada

**Affiliations:** Department of Gastrointestinal Oncology, Center for Adult Diseases, Osaka, Japan.

## Abstract

Ovariectomy at 1 month of age promotes development of hepatocellular adenomatous nodules in female C57BL/6 x DS-F1 mice treated neonatally with 3'-methyl-4-dimethylaminoazobenzene (3'-Me-DAB). Implantation of oestradiol-17 beta (E2) pellets at 1 month of age suppresses nodule development. Since E2 increases serum levels of prolactin, high serum levels of prolactin in mice that have received implants of E2 pellets may play a role in the suppression of hepatocellular tumorigenesis. Therefore, to investigate the role of prolactin in hepatocellular tumorigenesis, we examined development of adenomatous nodules in female mice that had been treated neonatally with 3'-Me-DAB and had undergone ovariectomy at 1 month of age, under various serum levels of prolactin. Treatment of these mice with perphenazine (dopamine antagonist) from 6 months of age or transplantation of pituitary glands under the renal capsule at 6 months of age markedly increased serum levels of prolactin and significantly suppressed the incidence of adenomatous nodules at 12 months of age. Implantation of E2 pellets at 1 month of age increased serum levels of prolactin to a greater extent and further decreased the incidence of adenomatous nodules. Treatment of mice that had received implants of E2 pellets at 1 month of age with bromocriptine (dopamine agonist) from 6 months of age decreased serum levels of prolactin, and was accompanied by an increase in the incidence of nodules. The present results showed that an increase in serum levels of prolactin was accompanied by a decrease in incidence of liver tumours induced by 3'-Me-DAB in mice, suggesting a suppressive effect of prolactin on liver tumorigenesis in mice. Thus, it is possible that the suppressive effect of oestrogen on liver tumorigenesis in mice is mediated, at least in part, by prolactin.


### Correlation between serum prolactin levels and hepatocellular tumorigenesis induced by 3′-methyl-4-dimethylaminoazobenzene in mice

R Yamamoto[1], H Iishi[1], M Tatsuta[1], T Yamamoto[2], K Koike[3], Y Kanda[3], A Miyake[3], M Tsuji[4] and N Terada[2]

*Departments of [1]Gastrointestinal Oncology and [2]Pathology, The Center for Adult Diseases, Osaka, 3 Nakamichi 1-chome, Higashinari-ku, Osaka 537; [3]Department of Obstetrics and Gynecology, Osaka University Medical School, Suita, Osaka 565; [4]Department of Pathology, Itami City Hospital, Itami, Hyogo 644, Japan.*

**Summary** Ovariectomy at 1 month of age promotes development of hepatocellular adenomatous nodules in female C57BL/6 × DS-$F_1$ mice treated neonatally with 3′-methyl-4-dimethylaminoazobenzene (3′-Me-DAB). Implantation of oestradiol-17β ($E_2$) pellets at 1 month of age suppresses nodule development. Since $E_2$ increases serum levels of prolactin, high serum levels of prolactin in mice that have received implants of $E_2$ pellets may play a role in the suppression of hepatocellular tumorigenesis. Therefore, to investigate the role of prolactin in hepatocellular tumorigenesis, we examined development of adenomatous nodules in female mice that had been treated neonatally with 3′-Me-DAB and had undergone ovariectomy at 1 month of age, under various serum levels of prolactin. Treatment of these mice with perphenazine (dopamine antagonist) from 6 months of age or transplantation of pituitary glands under the renal capsule at 6 months of age markedly increased serum levels of prolactin and significantly suppressed the incidence of adenomatous nodules at 12 months of age. Implantation of $E_2$ pellets at 1 month of age increased serum levels of prolactin to a greater extent and further decreased the incidence of adenomatous nodules. Treatment of mice that had received implants of $E_2$ pellets at 1 month of age with bromocriptine (dopamine agonist) from 6 months of age decreased serum levels of prolactin, and was accompanied by an increase in the incidence of nodules. The present results showed that an increase in serum levels of prolactin was accompanied by a decrease in incidence of liver tumours induced by 3′-Me-DAB in mice, suggesting a suppressive effect of prolactin on liver tumorigenesis in mice. Thus, it is possible that the suppressive effect of oestrogen on liver tumorigenesis in mice is mediated, at least in part, by prolactin.

**Keywords:** prolactin; 3′-methyl-4-dimethylaminoazobenzene; oestrogen

Administration of carcinogens to prepubertal mice induces development of hepatocellular tumours. Male mice are more susceptible than females (Klein and Weisburger, 1966; Vesselinovitch and Mihailovich, 1967; Vesselinovitch, 1969; Roe *et al.*, 1971; Vesselinovitch *et al.*, 1972, 1980; Rao and Vesselinovitch, 1973; Moore *et al.*, 1981; Kemp *et al.*, 1989). This sex difference in susceptibility is due in part to the promotive effect of androgens secreted by the testes after puberty (Vesselinovitch *et al.*, 1980; Moore *et al.*, 1981; Kemp *et al.*, 1989; Weghorst and Klaunig, 1989). Furthermore, several studies (Vesselinovitch and Mihailovich, 1967; Vesselinovitch *et al.*, 1980; Goldfarb and Pugh, 1990; Yamamoto *et al.*, 1991; Tsutsui *et al.*, 1992) have shown that ovariectomy after administration of carcinogens shortens the latent period in the development of hepatocellular tumours and increases their incidence, indicating that the ovaries suppress hepatocellular tumorigenesis. These findings suggest that an ovarian hormone, oestrogen or progesterone, suppresses hepatocellular tumorigenesis in mice.

Administration of 3′-methyl-4-dimethylaminoazobenzene (3′-Me-DAB) to neonatal female mice induces hepatocellular tumorigenesis (Roe *et al.*, 1971; Yamamoto *et al.*, 1991), and ovariectomy promotes development of hepatocellular tumours (Yamamoto *et al.*, 1991, 1993*a*; Tsutsui *et al.*, 1992). We have shown that the ovarian hormone oestradiol-17β ($E_2$) suppresses hepatocellular tumorigenesis, but that the other ovarian hormone, progesterone, does not (Yamamoto *et al.*, 1991, 1993*b*). Moreover, we suggested that the suppressive action of $E_2$ is due not to its direct action on the liver but to its indirect action on tissues other than the liver (Yamamoto *et al.*, 1993*b*).

Since oestrogen increases secretion of prolactin by the pituitary gland (Chen *et al.*, 1970; Meites, 1974), and since mouse liver contains prolactin receptors (Harigaya *et al.*, 1988; Davis and Linzer, 1989), it is conceivable that prolactin is an extrahepatic mediator of oestrogen's suppression of hepatocellular tumorigenesis. Therefore, to investigate the role of prolactin in hepatocellular tumorigenesis in mice, we examined hepatocellular tumorigenesis induced by 3′-methyl-4-dimethylaminoazobenzene in mice under various serum levels of prolactin. We produced high serum levels of prolactin by daily injections of perphenazine, a dopamine antagonist (Wicha *et al.*, 1980; Shinha and Gilligan, 1982; Singtripop *et al.*, 1991), or transplantation of pituitary glands under the renal capsule (Chen *et al.*, 1970; Lam *et al.*, 1976), and decreased the oestrogen-induced high serum levels of prolactin by daily injections of bromocriptine, a dopamine agonist (Mori and Nagasawa, 1984; Wood *et al.*, 1991).

Correspondence: R Yamamoto
Received 11 November 1994; revised 16 February 1995; accepted 21 February 1995.

### Materials and methods

### *Mice*

Female C57BL/6 × DS-$F_1$ mice (bred in our laboratory) were housed at 25°C under controlled lighting (12 h light/12 h darkness) and allowed free access to water and food pellets. Ovariectomy was performed under pentobarbital sodium anaesthesia.

### *Administration of carcinogen*

The carcinogen, 3′-Me-DAB (ICN Pharmaceuticals, Plainview, NY, USA) was suspended in an aqueous solution of 0.7% (w/v) gelatin at a concentration of 10 mg $ml^{-1}$; 0.05 ml of the suspension was injected intraperitoneally into mice 10, 12, 14, 16 and 18 days old.

### *Implantation of oestradiol-17β*

Cylindrical cholesterol pellets containing 1% (w/v) $E_2$ were prepared. Ten milligram pellets were implanted subcutaneously (s.c.) in the interscapular space. Pellets were replaced every 3 months.

### *Injection of perphenazine or bromocriptine*

Perphenazine (Sigma, St Louis, MO, USA) was dissolved in saline at a concentration of 1 mg $ml^{-1}$; 0.1 ml of the solution was injected s.c. daily. Bromocriptine (2-bromo-α-ergocriptine methanesulphonate salt) was dissolved in 10% (v/v) ethanol in saline at a concentration of 1 mg $ml^{-1}$; 0.1 ml of the solution was injected s.c. daily. Bromocriptine was kindly supplied by Sandoz Pharmaceuticals (Tokyo, Japan).

### *Transplantation of pituitary glands*

Four pituitary glands obtained from 5 to 6-month-old female C57BL/6 × DS-$F_1$ mice that had received no treatments were transplanted under the kidney capsule of 6-month-old mice that had been treated with 3′-Me-DAB neonatally and undergone ovariectomy at 1 month of age.

### *Treatment of mice*

The study consisted in two experiments (experiments I and II). All mice (318 mice) used in the experiments were treated neonatally with 3′-Me-DAB as described above, and underwent ovariectomy at 1 month of age. In experiment I (Figure 1), the mice were divided into four groups (groups 1, 2, 3 and 4). Group 1 mice ($n = 35$) received daily injections of saline (0.1 ml) from the age of 6 months. Group 2 mice ($n = 50$) received daily injections of perphenazine (0.1 mg) dissolved in 0.1 ml of saline. Group 3 mice ($n = 48$) received transplants of four pituitary glands under the kidney capsule at 6 months of age. Group 4 mice ($n = 53$) received implants of $E_2$ pellets (10 mg) at 1 month of age. In experiment II (Figure 2), the mice were divided into three groups (groups 1, 2 and 3). Group 1 mice ($n = 54$) received daily injections of 0.1 ml of vehicle (10% ethanol in saline) from the age of 6 months. Both groups 2 and 3 received implants of $E_2$ pellets (10 mg) at 1 month of age. In addition, group 2 mice ($n = 38$) received daily injections of bromocriptine (0.1 mg) dissolved in 0.1 ml of vehicle, while group 3 mice ($n = 40$) received injections of vehicle (0.1 ml) only from the age of 6 months. At 12 months of age, blood was taken from the inferior vena cava of all mice under pentobarbital sodium anaesthesia, after which the mice were killed by cervical dislocation and their livers were promptly removed.

In experiment I, we examined the effects on hepatocellular tumorigenesis of high serum levels of prolactin, produced by either daily injections of perphenazine (Wicha *et al*., 1980; Shinha and Gilligan, 1982; Singtripop *et al*., 1991) or transplantation of pituitary glands (Chen *et al*., 1970; Lam *et al*., 1976) (Figure 1).

In experiment II, we investigated the effects of bromocriptine, which decreases serum levels of prolactin (Mori and Nagasawa, 1984; Wood *et al*., 1991), on the suppressive action of oestrogen on hepatocellular tumorigenesis (Figure 2). Since our previous studies suggested that oestrogen exerts its suppressive effect after 6 months of age (Tsutsui *et al*., 1992; Yamamoto *et al*., 1993*a*), injections of perphenazine or bromocriptine were started and pituitary glands were transplanted at that time.

### *Histological examination of the liver*

The liver was fixed in Zamboni's solution and cut into 4-mm-thick serial strips. One section (5 μm) of each strip was stained with haematoxylin and eosin; all such sections were examined for nodular lesions, i.e. adenomatous nodules and carcinomas. An adenomatous nodule of hepatocellular origin was defined with reference to previous reports (Vesselinovitch *et al*., 1978; Frith *et al*., 1980; Lipsky *et al*., 1981) as described previously (Yamamoto *et al*., 1991) as a mixture of eosinophilic, basophilic, vacuolated and foamy hepatocytes in various proportions that compresses the adjacent parenchyma but does not contain a carcinomatous lesion with a trabecular structure. Hepatocellular carcinoma was defined as a nodular lesion with a trabecular structure, as described previously (Yamamoto *et al*., 1991).

**Figure 1** Design of experiment I. All female mice were treated with 3′-Me-DAB neonatally and underwent ovariectomy at 1 month of age. Groups 1 and 2 received daily injections of saline (0.1 ml) and perphenazine (0.1 mg in 0.1 ml of saline) from the age of 6 months. Group 3 received transplants of four pituitary glands under the kidney capsule at 6 months of age. Group 4 received implants of $E_2$ pellets (10 mg) at 1 month of age.

**Figure 2** Design of experiment II. All female mice were treated with 3′-Me-DAB neonatally and underwent ovariectomy at 1 month of age. Group 1 received daily injections with 0.1 ml of vehicle (10% ethanol in saline) from the age of 6 months. Groups 2 and 3 received implants of $E_2$ pellets at 1 month of age. Group 2 received daily injections of bromocriptine (0.1 mg in 0.1 ml of vehicle), and group 3 received vehicle (0.1 ml) only from 6 months of age.

### *Number of adenomatous nodules per mouse*

All sections of the liver prepared as described above were examined, and the number of adenomatous nodules was counted. An adenomatous nodule found in two adjacent sections was counted as one lesion.

### *Assay of serum prolactin*

Serum was obtained by centrifugation of blood at 1000 *g* for 10 min, and was stored at −80°C until assay. Serum prolactin was determined by double-antibody radioimmunoassay with materials and protocols supplied by AF Parlow (Pituitary Hormones and Antisera Center, Harbor-UCLA Medical Center, Torrance, CA, USA). Results are expressed in terms of standard AFP6476C. All samples were assayed in duplicate. The intra-assay and inter-assay variations were less than 8% and 10% respectively.

*Statistical analysis*

Statistical analysis was performed with the $\chi^2$ test or Student's *t*-test. A *P*-value below 0.05 was considered significant.

### Results

Table I shows the effects of perphenazine or transplantation of pituitary glands on development of adenomatous nodules induced by neonatally administered 3'-Me-DAB. The incidence of adenomatous nodules in mice that had undergone ovariectomy at 1 month of age was 71.4% at 12 months of age. Treatment with perphenazine from 6 months of age and transplantation of pituitary glands at 6 months of age significantly decreased the incidence of adenomatous nodules to 16.0% and 20.6% respectively, while implantation of $E_2$ pellets at 1 month of age decreased the incidence to 1.9%. However, the mean number of adenomatous nodules per mouse was not significantly affected by treatment with perphenazine or transplantation of pituitary glands. Treatment with perphenazine and transplantation of pituitary glands markedly elevated serum levels of prolactin, and implantation of $E_2$ pellets raised prolactin levels to an even greater extent.

Table II shows the effects of bromocriptine on development of adenomatous nodules in mice that had received implants of $E_2$ pellets. Implantation of $E_2$ pellets at 1 month of age markedly decreased the incidence of adenomatous nodules at 12 months of age to 5.0%, while the incidence was 66.7% in mice that did not receive $E_2$ pellets. When mice that had received $E_2$ pellets were treated with bromocriptine from 6 months of age, the incidence of adenomatous nodules increased to 23.7%. This incidence was significantly lower than that in mice that did not receive $E_2$ pellets, and was similar to that in mice treated with perphenazine or transplanted pituitary glands, as shown in Table I. The number of adenomatous nodules was not significantly affected by either implantation of $E_2$ pellets or treatment with bromocriptine. Serum prolactin levels in mice that received $E_2$ pellets were extremely high, in agreement with data shown in Table I. Treatment with bromocriptine significantly decreased serum prolactin to levels similar to those in mice treated with perphenazine or pituitary gland transplants, as shown in Table I. No carcinomas were found in any groups. No adenomatous nodules or carcinomas developed in the livers of 21 female mice at 12 months of age which had not been treated neonatally with 3'-Me-DAB, but had instead undergone ovariectomy at 1 month of age.

### Discussion

Increases in serum levels of prolactin produced by injections of perphenazine (dopamine antagonist) and by pituitary grafts were accompanied by decreases in the incidence of adenomatous nodules. The greater the increase in serum levels of prolactin produced by implantation of $E_2$ pellets, the greater was the decrease in the incidence of nodules. Furthermore, bromocriptine (dopamine agonist) decreased the high serum levels of prolactin induced by implantation of $E_2$ pellets and increased the incidence of adenomatous nodules. Dopamine agonists and antagonists produce changes in serum levels of prolactin (Shinha and Gilligan, 1982; Mori and Nagasawa, 1984; Singtripop *et al.*, 1991; Wood *et al.*, 1991), but have also been reported to exert other effects. For example, it is reported that dopamine antagonists stimulate aldosterone and corticosterone secretion in rats (Goebel *et al.*, 1992) and that bromocriptine increases serum levels of growth hormone in normal human subjects (Wood *et al.*, 1991). Pituitary grafts secrete growth hormone as well as prolactin (Blanck *et al.*, 1984). Moreover, a recent report by

**Table I** Effects of perphenazine or transplantation of pituitary glands on serum prolactin levels and development of adenomatous nodules in the liver

| *Treatment* | *Adenomatous nodules* *Incidence* | *Number per mouse*[a] | *Serum prolactin (ng ml$^{-1}$)*[a] | |
|---|---|---|---|---|
| Saline | 25/35 (71.4%)[b] | 2.5 ± 0.4 | 16.6 ± 3.3[c] | (*n* = 11) |
| Perphenazine | 8/50 (16.0%)[b,d] | 1.5 ± 0.3 | 178.6 ± 17.2[c,e] | (*n* = 20) |
| Transplantation of pituitary glands | 10/48 (20.6%)[b,d] | 1.4 ± 0.2 | 203.0 ± 22.2[c,e] | (*n* = 17) |
| Implantation of $E_2$ pellets | 1/53 (1.9%)[d] | 1.0 | 1320.5 ± 158.9[e] | (*n* = 15) |

The experimental design is shown in Figure 1. Serum concentrations of prolactin were measured in mice, the number of which is shown in parentheses. Incidence indicates the number of mice with adenomatous nodules out of number of mice examined, and percentage incidence is shown in parentheses. Number per mouse indicates the number of adenomatous nodules in each mouse with adenomatous nodules. [a]Mean ± s.e. [b,c]$P<0.05$, significant difference from the value of mice implanted with $E_2$ pellets ([b]by $\chi^2$ test; [c]by Student's *t*-test). [d,e]$P<0.05$, significant difference from the value of mice injected with saline alone ([d]by $\chi^2$ test; [e]by Student's *t*-test).

**Table II** Effects of bromocriptine injections on serum prolactin levels and development of adenomatous nodules

| *Treatment* | *Adenomatous nodules* *Incidence* | *Number per mouse*[a] | *Serum prolactin (ng ml$^{-1}$)*[a] | |
|---|---|---|---|---|
| Vehicle | 36/54 (66.7%)[b] | 1.9 ± 0.3 | 14.9 ± 3.1[c] | (*n* = 20) |
| Bromocriptine and implantation of $E_2$ pellets | 9/38 (23.7%)[b,d] | 1.3 ± 0.2 | 164.5 ± 21.0[c,e] | (*n* = 15) |
| Implantation of $E_2$ pellets | 2/40 (5.0%)[d] | 1,2 | 818.5 ± 158.1[e] | (*n* = 15) |

The experimental design is shown in Figure 2. Serum concentrations of prolactin were measured in mice, the number of which is shown in parentheses. Incidence indicates the number of mice with adenomatous nodules out of number of mice examined, and percentage incidence is shown in parentheses. Number per mouse indicates the number of adenomatous nodules in each mouse with adenomatous nodules. [a]Mean ± s.e. [b,c]$P<0.05$, significant difference from the value of mice implanted with $E_2$ pellets ([b]by $\chi^2$ test; [c]by Student's *t*-test). [d,e]$P<0.05$, significant difference from the value of mice injected with vehicle alone ([d]by $\chi^2$ test; [e]by Student's *t*-test).

Ishibashi et al. (1994) that bromocriptine inhibits growth of human small-cell lung cancer through tumour dopamine receptors raises the possibility that dopamine agonists and antagonists influence 3'-Me-DAB-induced tumorigenesis through a direct effect on hepatocytes. Therefore, we cannot exclude the possibility that the influences of perphenazine, pituitary grafts and bromocriptine on liver tumorigenesis are irrelevant to influences on serum levels of prolactin. However, our finding that the increase in serum levels of prolactin was accompanied by a suppression of liver tumorigenesis in mice supports the possibility that high serum levels of prolactin suppress development of 3'-Me-DAB-induced hepatocellular tumours in mice. If this is true, then it is likely that the suppressive action of oestrogen on hepatocellular tumorigenesis in mice is mediated, at least in part, by prolactin secreted by the pituitary glands.

In rats prolactin has been shown to induce hepatic ornithine decarboxylase and plasminogen activator activity and specific enzyme markers expressed early in the $G_1$ phase of the cell cycle (Crowe et al., 1991), to cause hypomethylation of DNA in the liver (Reddy and Reddy, 1990), to stimulate DNA synthesis by hepatocytes (Buckley et al., 1986) and to produce hepatomegaly (Buckley et al., 1985). Furthermore, it is reported that prolactin promotes development of diethylnitrosamine-induced preneoplastic γ-glutamyltranspeptidase-positive foci in the liver of female rats (Buckley et al., 1985). Blank et al. (1987) reported that prolactin did not suppress growth in male rats of γ-glutamyltransferase-positive hepatic foci induced by diethylnitrosamine followed by treatment with 2-acetylaminofluorene. Thus, the effects of prolactin on the development of carcinogen-induced hepatocellular tumours in rats may be opposite to those in mice, although it is unknown whether the effects of prolactin on normal hepatocytes differ in mice and rats.

The effects of oestrogens on hepatocellular tumorigenesis in rats are opposite to those in mice. In mice, a natural oestrogen, $E_2$, and a synthetic oestrogen, ethinyloestradiol, have been reported to suppress carcinogen-induced hepatocellular tumorigenesis (Lee et al., 1989; Yamamoto et al., 1993a,b). In contrast, in rats several synthetic oestrogens have been shown to promote carcinogen-induced hepatocellular tumorigenesis (Metzler and Degen, 1987; Campen et al., 1990; Mayol et al., 1992). A synthetic oestrogen has been shown to increase serum levels of prolactin (Campen et al., 1990). Synthetic oestrogens may exert toxic effects on the liver that contribute to the promotion of hepatocellular tumorigenesis (Metzler and Degen, 1987). However, if the promotive effects of synthetic oestrogens on hepatocellular tumorigenesis are due to the high serum levels of prolactin which they produce, the difference in the effects of oestrogens on hepatocellular tumorigenesis in mice and rats might be due to the difference in the effects of prolactin on hepatocellular tumorigenesis.

In carcinogen-induced hepatocellular tumorigenesis in rats, the difference in the secretory pattern of growth hormone is due, at least in part, to the lesser susceptibility of females to carcinogens; higher basal levels of serum growth hormone in females suppresses hepatocellular tumorigenesis (Blank et al., 1987; Hallstrom et al., 1991). The role of growth hormone in suppression of hepatocellular tumorigenesis in female mice is unknown, but the present results suggest that the pituitary gland plays a role in hepatocellular tumorigenesis in mice.

**Acknowledgements**
This work has been supported in part by grants from the Association for Prevention of Adult Diseases and the Osaka Cancer Foundation.

**References**

BLANK A, HANSSON T, ERIKSSON LC AND GUSTAFSSON J-Å. (1984). On mechanisms of sex differences in chemical carcinogenesis: effects of implantation of ectopic pituitary grafts on the early stages of liver carcinogenesis in the rat. *Carcinogenesis*, **5**, 1257–1262.

BLANK A, HANSSON T, ERIKSSON LC AND GUSTAFSSON J-Å. (1987). Growth hormone modifies the growth rate of enzyme-altered hepatic foci in male rats treated according to the resistant hepatocyte model. *Carcinogenesis*, **18**, 1585–1588.

BUCKLEY AR, PUTNAM CW AND RUSSELL DH. (1985). Prolactin is a tumor promoter in rat liver. *Life Sci.*, **37**, 2569–2575.

BUCKLEY AR, PUTNAM CW, MONTGOMERY DW AND RUSSELL DH. (1986). Prolactin administration stimulates rat hepatic DNA synthesis. *Biochem. Biophys. Res. Commun.*, **138**, 1138–1145.

CAMPEN D, MARONPOT R AND LUCIER G. (1990). Dose–response relationships in promotion of rat hepatocarcinogenesis by 17α-ethinylestradiol. *J. Toxicol. Environ. Health*, **29**, 257–268.

CHEN CL, AMENOMORI Y, LU KH, VOOGT JL AND MEITES J. (1970). Serum prolactin levels in rats with pituitary transplants or hypothalamic lesions. *Neuroendocrinology*, **6**, 220–227.

CROWE PD, BUCKLEY AR, ZORN NE AND RUI H. (1991). Prolactin activates protein kinase C and stimulates growth-related gene expression in rat liver. *Mol. Cell. Endocrinol.*, **79**, 29–35.

DAVIS JA AND LINZER DIH. (1989). Expression of multiple forms of the prolactin receptor in mouse liver. *Mol. Endocrinol.*, **3**, 674–680.

FRITH GH, BAETCKE KP, NELSON CJ AND SCHIEFERSTEIN G. (1980). Sequential morphogenesis of liver tumors in mice given benzidine dihydrochloride. *Eur. J. Cancer*, **16**, 1205–1216.

GOEBEL S, DIETRICH M, JARRY H AND WUTTKE W. (1992). Indirect evidence to suggest that prolactin mediates the adrenal action of haloperidol to stimulate aldosterone and corticosterone secretion in rats. *Endocrinology*, **130**, 914–919.

GOLDFARB S AND PUGH TD. (1990). Ovariectomy accelerates the growth of microscopic hepatocellular neoplasms in the mouse: possible association with whole body growth and fat disposition. *Cancer Res.*, **50**, 6779–6782.

HALLSTROM IP, SVENSSON D AND BLANCK A. (1991). Sex-differentiated deoxycholic acid promotion of rat liver carcinogenesis is under pituitary control. *Carcinogenesis*, **12**, 2035–2040.

HARIGAYA T, SMITH WC AND TALAMANTES F. (1988). Hepatic placental lactogen receptors during pregnancy in the mouse. *Endocrinology*, **122**, 1366–1372.

ISHIBASHI M, FUJISAWA M, FURUE H, MAEDA Y, FUKAYAMA M AND YAMAJI T. (1994). Inhibition of growth of human small cell lung cancer by bromocriptine. *Cancer Res.*, **54**, 3442–3446.

KEMP CJ, LEARY CN AND DRINKWATER NR. (1989). Promotion of murine hepatocarcinogenesis by testosterone is androgen receptor-dependent but not cell autonomous. *Proc. Natl Acad. Sci. USA*, **86**, 7505–7509.

KLEIN M AND WEISBURGER EK. (1966). Carcinogenic effect of N-hydroxy-N-2-fluorenylacetamide, 2'4'-dimethylacetanilide, and 2'4'6'-trimethylacetanilide on liver in suckling mice. *Proc. Soc. Exp. Biol. Med.*, **122**, 111–114.

LAM PCO, MORISHIGE WK AND ROTHCHILD I. (1976). Venous outflow of the hormones secreted by the rat pituitary autotransplanted beneath the kidney capsule. *Proc. Soc. Exp. Biol. Med.*, **152**, 615–617.

LEE GH, NAMURA K AND KITAGAWA T. (1989). Comparative study of diethylnitrosamine-induced two-stage hepatocarcinogenesis in C3H, C57BL and BALB mice promoted by various hepatopromoters. *Carcinogenesis*, **10**, 2227–2230.

LIPSKY MM, HINTON DE, KLAUNIG JE AND TRUMP BF. (1981). Biology of hepatocellular neoplasia in the mouse. I. Histogenesis of safrole-induced hepatocellular carcinoma. *J. Natl Cancer Inst.*, **67**, 365–376.

MAYOL X, NEAL GE, DAVIES R, ROMERO A AND DOMINGO J. (1992). Ethinyl estradiol-induced cell proliferation in the rat liver. Involvement of specific populations of hepatocytes. *Carcinogenesis*, **12**, 2381–2388.

MEITES J. (1974). Relation of estrogen to prolactin secretion in animals and man. *Adv. Biosci.*, **4**, 195–208.

METZLER M AND DEGEN GH. (1987). Sex hormones and neoplasia: liver tumors in rodents. *Arch. Toxicol. Suppl.*, **10**, 251-263.

MOORE MR, DRINKWATER NR, MILLER EC, MILLER JA AND PITOT HC. (1981). Quantitative analysis of the time-dependent development of glucose-6-phosphatase-deficient foci in the liver of mice treated neonatally with diethylnitrosamine. *Cancer Res.*, **41**, 1585-1593.

MORI T AND NAGASAWA H. (1984). Alteration of the development of mammary hyperplastic alveolar nodules and uterine adenomyosis in SHN mice by different schedules of treatment with CB-154. *Acta Endocrinol.*, **107**, 245-249.

RAO KVN AND VESSELINOVITCH SD. (1973). Age- and sex-associated diethylnitrosamine dealkylation activity of the mouse liver and hepatocarcinogenesis. *Cancer Res.*, **33**, 1625-1627.

REDDY PMS AND REDDY PRK. (1990). Effect of prolactin on DNA methylation in the liver and kidney of rat. *Mol. Cell Biochem.*, **95**, 43-47.

ROE FJC, WARWICK GP, CARTER RL, PETO R, ROSS WCJ, MITCHLEY BCV AND BARRON NA. (1971). Liver and lung tumors in mice exposed at birth to 4-dimethylaminoazobenzene or its 2-methyl or 3'-methyl derivatives. *J. Natl Cancer Inst.*, **47**, 593-599.

SINGTRIPOP T, MORI T, PARK MK, SAKAMOTO S AND KAWASHIMA S. (1991). Development of uterine adenomyosis after treatment with dopamine antagonists in mice. *Life Sci.*, **49**, 201-206.

SHINHA YN AND GILLIGAN TA. (1982). Estrogen in high doses inhibits perphenazine-induced prolactin release. *Endocrinology*, **110**, 126-130.

TSUTSUI S, YAMAMOTO R, IISHI H, TATSUTA M, TSUJI M AND TERADA N. (1992). Promoting effect of ovariectomy on hepatocellular tumorigenesis induced in mice by 3'-methyl-4-dimethylaminoazobenzene. *Virchows Archiv. B Cell Pathol.*, **62**, 371-375.

VESSELINOVITCH SD. (1969). The sex-dependent difference in the development of liver tumors in mice administered dimethylnitrosamine. *Cancer Res.*, **29**, 1024-1027.

VESSELINOVITCH SD AND MIHAILOVICH N. (1967). The effect of gonadectomy on the development of hepatomas induced by urethane. *Cancer Res.*, **27**, 1788-1791.

VESSELINOVITCH SD, MIHAILOVICH N, WAGAN GN, LOMBARD LS AND RAO KVN. (1972). Aflatoxin $B_1$, a hepatocarcinogen in the infant mouse. *Cancer Res.*, **32**, 2282-2291.

VESSELINOVITCH SD, MIHAILOVICH N AND RAO KVN. (1978). Morphology and metastatic nature of induced hepatic nodular lesions in C57BL × C3H $F_1$ mice. *Cancer Res.*, **38**, 2003-2010.

VESSELINOVITCH SD, ITZE L, MIHAILOVICH N AND RAO KVN. (1980). Modifying role of partial hepatectomy and gonadectomy in ethylnitrosourea-induced hepatocarcinogenesis. *Cancer Res.*, **40**, 1538-1542.

WEGHORST CM AND KLAUNIG JE. (1989). Phenobarbital promotion in diethylnitrosamine-induced infant B6C3F1 mice: influence of gender. *Carcinogenesis*, **10**, 609-612.

WICHA MS, LIOTTA BK, VONDERHAAR BK AND KIDWELL WR. (1980). Effects of inhibition of basement membrane collagen deposition on rat mammary gland development. *Devel. Biol.*, **80**, 235-266.

WOOD DF, JOHNSTON JM AND JOHNSTON DJ. (1991). Dopamine, the dopamine D2 receptor and pituitary tumours. *Clin. Endocr.*, **35**, 455-466.

YAMAMOTO R, IISHI H, TATSUTA M, TSUJI M AND TERADA N. (1991). Roles of ovaries and testes in hepatocellular tumorigenesis induced in mice by 3'-methyl-4-dimethylaminoazobenzene. *Int. J. Cancer*, **49**, 83-88.

YAMAMOTO R, IISHI H, TATSUTA M, TSUJI M AND TERADA N. (1993*a*). Suppressive effect of estrogen on hepatocellular tumorigenesis induced in mice by 3'-methyl-4-dimethylaminoazobenzene. *Exp. Toxicol. Pathol.*, **45**, 325-328.

YAMAMOTO R, TATSUTA M AND TERADA N. (1993*b*). Suppression by oestrogen of hepatocellular tumorigenesis induced in mice by 3'-methyl-4-dimethylaminoazobenzene. *Br. J. Cancer*, **68**, 303-307.